# Histologically Confirmed Celiac Disease in a Multifactorial Primary-Care Presentation with Psychiatric, Musculoskeletal, and Hepatic Findings: A Case Report

**DOI:** 10.3390/jcm15145448

**Published:** 2026-07-12

**Authors:** Tomasz Karczewski, Dawid Karczewski

**Affiliations:** Cranston Ridge Medical Clinic, Calgary, AB T3M 3A9, Canada; tomasz@cranstonridgemedical.com

**Keywords:** celiac disease, histologically confirmed celiac disease, anxiety, depression, primary care, case report, extraintestinal manifestations, liver enzymes, hyperferritinemia, hypothyroidism, hepatic steatosis, gluten-free diet, tTG-IgA, duodenal biopsy

## Abstract

**Background/Objectives:** Celiac disease (CD) is an immune-mediated enteropathy with gastrointestinal and extraintestinal manifestations. In primary care, recognition can be delayed when psychiatric symptoms, arthralgia, thyroid dysfunction, alcohol exposure, and liver-test abnormalities coexist. This case report describes a confounder-aware diagnostic approach to histologically confirmed CD in a patient with a multifactorial primary-care presentation. **Methods:** We report a single-patient, de-identified reflective case from routine family medicine practice, organized according to CARE case-report principles. **Results:** A woman in her early sixties with hypothyroidism and glaucoma presented with new low mood, anhedonia, somnolence, generalized anxiety, increased alcohol intake, poor appetite, weight loss, abdominal bloating, diarrhea, flatulence, and polyarthralgia. Initial investigations, including celiac serology obtained before gluten-free diet advice, showed mild anemia, marked hyperferritinemia, severe cholestatic and hepatocellular liver-test abnormalities, uncontrolled hypothyroidism, and strongly positive tissue transglutaminase IgA (>250 kIU/L; reference 0.0–14.9). Radiographs showed mild osteoarthritis and osteopenia without erosive arthropathy. Computed tomography excluded malignancy but showed severe diffuse hepatic steatosis and mild pancreatic atrophy. Mirtazapine was started at the index visit; after the initial laboratory results, gluten-free diet advice, alcohol-reduction counseling, and levothyroxine adjustment were undertaken. During the diagnostic episode, small-bowel biopsy demonstrated moderate-to-severe crypt hyperplastic villous atrophy with increased intraepithelial lymphocytes, and gastric biopsies showed no significant pathology; the histology was consistent with CD. Symptoms improved substantially. Longer-term objective follow-up showed persistent but improved celiac serology (tTG-IgA 49.4 kIU/L), normalization of thyroid-stimulating hormone, partial improvement in gamma-glutamyl transferase, which remained elevated, and a later iron-deficiency pattern with persistent anemia. **Conclusions:** This case supports targeted CD testing when anxiety or depressive symptoms occur alongside gastrointestinal symptoms, weight loss, arthralgia, hypothyroidism or documented thyroid autoimmunity, anemia, osteopenia, or liver-test abnormalities. Histology and repeat serology confirmed the diagnosis, but the psychiatric and hepatic manifestations still require cautious interpretation because hypothyroidism, alcohol exposure, steatotic liver disease, and simultaneous treatments also shaped the clinical course.

## 1. Introduction

Celiac disease (CD) is a chronic immune-mediated enteropathy triggered by gluten exposure in genetically predisposed individuals. Although CD has traditionally been associated with diarrhea, malabsorption, weight loss, and nutritional deficiency, contemporary guidelines and reviews emphasize its broader multisystem phenotype, including gastrointestinal, hematologic, hepatic, musculoskeletal, endocrine, neurologic, and psychiatric manifestations [1,2,3,4]. Population-based studies estimate that CD affects approximately 1% of many populations, although prevalence varies by geography, age, sex, method of ascertainment, and whether diagnosis is based on serology, biopsy, or both [2,3].

The primary-care challenge is that nonclassical presentations rarely arrive as a single diagnostic label. Patients may present first with fatigue, mood symptoms, sleep disturbance, arthralgia, abnormal liver biochemistry, anemia, osteopenia, or autoimmune comorbidity. Psychiatric manifestations have been reported in association with CD, including depression and anxiety, but the direction of association and mechanisms remain incompletely defined [5]. Liver biochemical abnormalities are also described in CD, and some cases improve after a strict gluten-free diet (GFD); however, abnormal liver tests have a broad differential, and competing etiologies such as alcohol-related liver injury, steatotic liver disease, viral hepatitis, autoimmune liver disease, biliary disease, medication effects, thyroid dysfunction, and iron overload must be considered [6].

This distinction is particularly important in patients with multiple plausible explanations for psychiatric or systemic symptoms. Overt hypothyroidism can contribute to fatigue, cognitive slowing, low mood, and reduced quality of life [7]. Autoimmune thyroid disease is also more common among patients with CD than in the general population, supporting targeted screening when compatible gastrointestinal or systemic features are present [8]. Therefore, the clinical task is not to attribute every symptom to CD, but to decide whether CD is sufficiently likely to justify testing while maintaining an appropriate differential diagnosis.

This case report describes an adult patient who presented to family medicine with new anxiety and depressive symptoms, gastrointestinal symptoms, weight loss, arthralgia, severe liver-test abnormalities, uncontrolled hypothyroidism, increased alcohol intake, severe hepatic steatosis, and strongly positive tTG-IgA. The initial tTG-IgA was obtained before gluten-free diet advice. The diagnosis was subsequently strengthened during the diagnostic episode by small-bowel histology consistent with CD and later by repeat serology showing persistent but improved tTG-IgA positivity. The purpose is educational: to reinforce targeted CD screening in primary care while showing how diagnostic certainty can improve through follow-up without losing sight of competing explanations for mood, liver, thyroid, and nutritional findings.

## 2. Case Presentation

### 2.1. Reporting Framework and De-Identification

This manuscript was prepared as a single-patient, de-identified reflective case report from routine primary care. The narrative follows core CARE case-report elements, including patient information, clinical findings, diagnostic assessment, intervention, follow-up, discussion, limitations, and consent considerations [9]. The case did not involve a prospective research protocol, randomization, experimental intervention, recruitment, or analysis of a patient cohort. Potentially identifying details have been minimized; for example, the patient age is presented as an age range rather than an exact age.

### 2.2. Patient Information and Presenting Concerns

A woman in her early sixties with a past medical history of hypothyroidism and glaucoma presented to her family physician because of deterioration in mental health. She described low mood, anhedonia, persistent somnolence, and high levels of generalized anxiety. Before seeking medical review, she had tried several over-the-counter or natural products, including vitamin B12, vitamin D3, and valerian drops. She and her family were also concerned that her alcohol consumption had increased.

During the same clinical encounter, she reported poor appetite, unintentional weight loss, abdominal bloating, diarrhea, and flatulence. She also described pain and swelling involving multiple joints, including the hands, ankles, and knees. These gastrointestinal, musculoskeletal, endocrine, hepatic, and psychiatric features prompted the treating physician to investigate beyond an isolated primary mental-health diagnosis.

### 2.3. Clinical Findings and Initial Investigations

A broad initial panel was requested because psychiatric symptoms occurred in the context of weight loss, gastrointestinal symptoms, polyarthralgia, and systemic features. Tests included complete blood count, electrolytes, C-reactive protein, iron studies, liver enzymes, thyroid-stimulating hormone (TSH), tTG-IgA, ferritin, fasting glucose, hemoglobin A1C, calcium, uric acid, lipid profile, and radiographs of the hands and knees. Mirtazapine 15 mg nightly was initiated for mood, sleep, and appetite symptoms. The baseline clinical, laboratory, imaging, and histological findings are summarized in Table 1.

### 2.4. Imaging, Diagnostic Assessment, and Differential Diagnosis

Radiographs of the hands and knees demonstrated mild osteoarthritis and mild osteopenia, without evidence of erosive or inflammatory arthropathy. Because of weight loss and markedly abnormal blood tests, computed tomography (CT) of the abdomen and pelvis was requested to exclude malignancy. CT abdomen/pelvis showed severe diffuse hepatic steatosis and mild pancreatic atrophy, with no evidence of malignancy. The imaging findings were clinically important: they helped exclude malignancy, but they also kept steatotic liver disease and possible alcohol-related hepatic injury in the differential diagnosis.

The strongly positive tTG-IgA result, obtained before the patient received gluten-free diet advice, substantially increased suspicion for CD in the setting of compatible gastrointestinal and extraintestinal symptoms. Subsequent small-bowel biopsies performed during the same diagnostic episode demonstrated moderate-to-severe crypt hyperplastic villous atrophy with increased intraepithelial lymphocytes, and gastric biopsies from the antrum/body showed no significant pathology. The pathology interpretation was consistent with CD. The available source record does not quantify day-to-day gluten exposure immediately before biopsy; however, the combination of compatible symptoms, strongly positive pre-diet serology, and small-bowel histology supports histologically confirmed CD. Adult diagnostic guidance supports diagnosis through integration of clinical features, celiac-specific serology, and duodenal histopathology while the patient remains on a gluten-containing diet [1,10]. The Oslo definitions remain useful for distinguishing confirmed disease from less certain categories when diagnostic evidence is incomplete or discordant [11].

The differential diagnosis nevertheless remained broad. Psychiatric symptoms could have reflected a primary depressive or anxiety disorder, but uncontrolled hypothyroidism, alcohol intake, sleep disturbance, chronic gastrointestinal symptoms, weight loss, and systemic illness were also plausible contributors. The liver-test pattern was too severe to attribute automatically to CD: increased alcohol intake, severe hepatic steatosis, metabolic or alcohol-associated steatotic liver disease, cholestatic or biliary pathology, viral hepatitis, autoimmune hepatopathy, medication effects, inflammatory disease, and iron overload all required consideration. Marked ferritin elevation was interpreted as a nonspecific but clinically important finding that could reflect liver injury, inflammation, alcohol exposure, metabolic disease, or iron overload rather than iron status alone [12]. The main differential diagnostic considerations and attribution issues are summarized in Table 2.

**Table 2:** Differential diagnosis and attribution considerations.

Finding	Supports Confirmed CD?	Important Competing or Coexisting Explanations	Clarification or Follow-Up Needed
Diarrhea, bloating, flatulence, weight loss	Yes; compatible gastrointestinal features and supported by serology and small-bowel histology.	Alcohol use, pancreatic disease, medication effects, malignancy, inflammatory bowel disease, functional bowel disorder.	Monitor symptoms and tTG-IgA response; confirm dietetic support, GFD adherence, and gluten exposure history around diagnostic testing.
Low mood, anxiety, anhedonia, somnolence	Possible association, but not diagnostic and not enough to infer causality.	Uncontrolled hypothyroidism, alcohol use, primary mood/anxiety disorder, sleep disturbance, medication or nutritional factors.	Repeat TSH/free T4, mental-health follow-up, alcohol history, medication response, standardized symptom measures if available.
Arthralgia and mild osteopenia	Can occur in CD or malabsorption-related bone disease.	Osteoarthritis, age-related osteopenia, thyroid disease, vitamin D deficiency, inflammatory arthropathy.	Vitamin D, calcium/phosphate, parathyroid hormone if indicated, bone-density assessment when appropriate.
Severe liver-test abnormalities and hepatic steatosis	CD can be associated with liver-test abnormalities, but the severity requires cautious attribution.	Alcohol-related liver injury, steatotic liver disease, biliary disease, viral hepatitis, autoimmune liver disease, medication injury.	Repeat full liver panel, synthetic function, platelet count, fibrosis assessment, viral and autoimmune testing, biliary evaluation, hepatology referral if persistent.
Ferritin 1538 microg/L at baseline	Nonspecific; may accompany inflammation or liver disease.	Alcohol exposure, steatotic liver disease, inflammation, infection, malignancy, iron overload.	Follow-up iron studies showed iron deficiency rather than overload at that point; continue monitoring and investigate anemia.
Known hypothyroidism with TSH 21.99 mIU/L	Thyroid autoimmunity is associated with CD, but autoimmune thyroiditis was not documented in this patient.	Medication nonadherence, undertreatment, malabsorption affecting levothyroxine, thyroid disease itself causing fatigue and mood symptoms.	Repeat TSH and free T4 after dose change; adherence review; thyroid peroxidase antibody only if autoimmune status is clinically relevant.

Abbreviations: CD, celiac disease; EMA-IgA, endomysial immunoglobulin A; GFD, gluten-free diet; HFE, homeostatic iron regulator gene; tTG-IgA, tissue transglutaminase immunoglobulin A; TSH, thyroid-stimulating hormone. The confounder-aware diagnostic and follow-up pathway for this case is shown in Figure 1.

**Figure 1:**
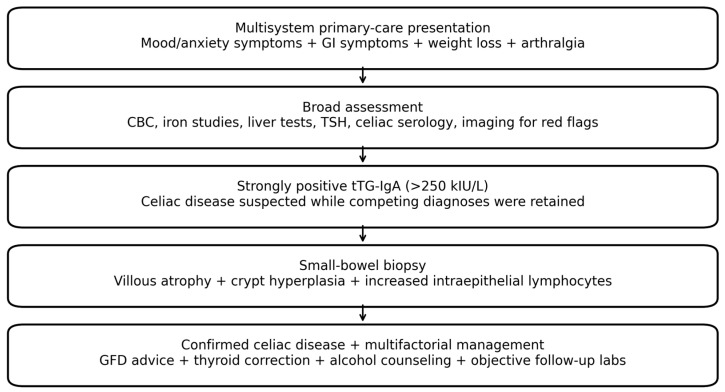
Confounder-aware diagnostic and follow-up pathway for this case.

### 2.5. Therapeutic Intervention

Mirtazapine 15 mg nightly was started at the index visit for mood, sleep, and appetite symptoms, before the initial laboratory results were available. After the baseline investigations returned, the patient was advised to begin a strict GFD and to stop or substantially reduce alcohol consumption. Her levothyroxine dose was adjusted to address the elevated TSH. Dietitian support and gastroenterology follow-up were recommended. Management continued in the community with emphasis on patient-centered counseling, adherence to a GFD, alcohol reduction, thyroid correction, and objective follow-up of serology, liver biochemistry, iron status, and anemia.

### 2.6. Follow-Up and Outcome

Over approximately six months after the initial management changes, the patient reported near-complete resolution of most symptoms. This clinical improvement is encouraging but still requires careful interpretation. Several interventions occurred close together: mirtazapine was started at the index visit, thyroid replacement was adjusted after baseline testing, alcohol-reduction counseling was provided, and a GFD was initiated after celiac serology had been obtained. Therefore, improvement in mood, appetite, sleep, gastrointestinal symptoms, and systemic well-being cannot be attributed solely to gluten withdrawal.

Longer-term objective follow-up results, available from March and May 2026, helped narrow diagnostic uncertainty and clarify ongoing monitoring needs. Repeat tTG-IgA in May 2026 remained positive at 49.4 kIU/L (reference <15.0), but this represented a substantial decline from the initial value of >250 kIU/L. Thyroid function improved, with follow-up TSH values of 0.94 and 0.36 mIU/L and free T4 of 15.7 pmol/L. GGT improved markedly to 104 U/L, although it remained above the reference range. Follow-up iron studies showed a shift from initial hyperferritinemia to an iron-deficiency pattern, with ferritin 8–15 microg/L, low iron, elevated total iron-binding capacity, and low transferrin saturation; anemia persisted, with hemoglobin reported at 95 g/L and later 110 g/L with microcytic features. Vitamin B12, vitamin D, CRP, renal function, and INR were reassuring, while hemoglobin A1C was elevated at 6.6% and lipase was mildly elevated. These results support CD as a confirmed diagnosis and document partial biochemical response, but they do not establish CD as the main cause of the initial liver abnormalities. Continued monitoring for liver disease, iron deficiency, metabolic risk, and thyroid disease remained necessary. The clinical course, including presentation, initial investigations, early management, histology, and longer-term follow-up, is summarized in Table 3.

The objective follow-up findings and their remaining clinical implications are summarized in Table 4.

## 3. Discussion

### 3.1. Educational Value for Primary Care

This case is best understood as an educational primary-care report rather than a description of a new clinical phenomenon. Its value lies in showing how a familiar diagnosis can be missed or delayed when psychiatric symptoms are the immediate reason for consultation and when multiple comorbidities compete for explanatory priority. The case reinforces an established diagnostic principle: CD should be considered when mental-health symptoms occur together with gastrointestinal symptoms, weight loss, anemia, hypothyroidism or documented autoimmune thyroid disease, arthralgia, osteopenia, or abnormal liver tests. The later pathology and repeat serology make the case more instructive because they show how follow-up can move a patient from diagnostic suspicion to confirmation.

The case also shows why real-world primary care often requires careful follow-up rather than a single decisive visit. At the first presentation, CD was only one part of a broad differential. Histology and repeat serology subsequently strengthened the diagnosis, while persistent anemia, iron deficiency, residual GGT elevation, hepatic steatosis, alcohol exposure, and corrected hypothyroidism continued to require separate clinical attention.

### 3.2. Diagnostic Certainty: Histology and Repeat Serology Narrowed the Differential

A markedly elevated tTG-IgA level obtained before gluten-free diet advice strongly supported CD, particularly because it was accompanied by diarrhea, bloating, weight loss, anemia, osteopenia, arthralgia, and thyroid disease. In this case, the diagnosis was strengthened further by small-bowel biopsy during the diagnostic episode showing moderate-to-severe crypt hyperplastic villous atrophy and increased intraepithelial lymphocytes, with the pathology report interpreting the findings as consistent with CD. This combination of compatible symptoms, markedly positive pre-diet serology, and supportive histology makes CD a confirmed diagnosis in this case.

The repeat tTG-IgA result also matters. A decline from >250 kIU/L to 49.4 kIU/L supports an improving celiac-specific immune signal, although the value remained positive and therefore still requires follow-up. Persistent positivity may reflect ongoing gluten exposure, incomplete mucosal recovery, variable adherence, or insufficient time for normalization. HLA-DQ2/DQ8 testing can be useful when serology and histology are discordant or when a patient has already started a GFD before adequate diagnostic testing. In this case, however, strongly positive serology obtained before GFD advice plus small-bowel histology consistent with CD makes HLA testing unlikely to add much diagnostic value [1,10,11].

### 3.3. Psychiatric Symptoms and Competing Explanations

Psychiatric manifestations of CD are increasingly discussed in the literature. Systematic review evidence has reported associations between CD and depression and anxiety, and prospective data suggest that quality of life and psychological scores may improve after diagnosis and treatment in some patients [5,13]. However, association does not establish causation. In the present case, the psychiatric presentation was plausibly multifactorial.

The patient had uncontrolled hypothyroidism, with a TSH of 21.99 mIU/L. Overt hypothyroidism can contribute to fatigue, cognitive slowing, apathy, low mood, and other neuropsychiatric complaints [7]. Follow-up TSH values normalized after levothyroxine adjustment, making thyroid correction a plausible contributor to improvement in somnolence, energy, and mood. Increased alcohol intake may also worsen mood, sleep, appetite, cognition, and liver injury. Mirtazapine was introduced at the first visit and may have improved sleep, appetite, anxiety, and depressive symptoms. For these reasons, the revised interpretation does not state that CD caused the psychiatric symptoms. Instead, CD is presented as one important diagnosis identified during investigation of a broader systemic and psychiatric presentation.

### 3.4. Liver-Test Abnormalities, Hepatic Steatosis, Pancreatic Atrophy, and Hyperferritinemia

The liver biochemistry in this case was severe, particularly the very high gamma-glutamyl transferase, elevated alkaline phosphatase, transaminase abnormalities, marked ferritin elevation, increased alcohol intake, and CT evidence of severe diffuse hepatic steatosis. CD can be associated with elevated aminotransferases and, less commonly, with autoimmune or cryptogenic liver disease; some abnormalities may improve after a GFD [6]. However, the severity and pattern here require caution. Alcohol-related liver injury and steatotic liver disease related to metabolic factors, alcohol exposure, or both are strong competing explanations and should not be treated as incidental.

Follow-up testing showed marked improvement in GGT to 104 U/L, but the value remained elevated. This supports biochemical improvement, yet it does not prove that CD was the main cause of the original liver abnormalities. Severe hepatic steatosis on CT and increased alcohol intake remain strong competing explanations. The CT finding of severe hepatic steatosis still needs to be integrated directly into the diagnostic reasoning. Contemporary liver terminology recognizes steatotic liver disease as an umbrella category that may include metabolic dysfunction-associated steatotic liver disease and alcohol-associated or mixed metabolic-and-alcohol-associated presentations [14]. In this patient, the available record does not provide enough detail to classify the liver disease subtype. Patients with celiac disease may also require longitudinal metabolic follow-up, as metabolic syndrome and fatty liver have been reported in celiac disease cohorts, including after initiation of a gluten-free diet [15]. Structured follow-up should include repeat full liver enzymes, synthetic liver function, platelet count, fibrosis risk stratification, alcohol history quantification, metabolic risk assessment, viral hepatitis testing, autoimmune liver screen, biliary evaluation when indicated, and consideration of hepatology referral [14,16].

The ferritin trajectory is also informative. The baseline ferritin of 1538 microg/L was not by itself evidence of iron overload because ferritin is also an acute-phase reactant and may rise with liver disease, alcohol exposure, metabolic disease, inflammation, infection, and malignancy [12]. Later follow-up showed ferritin in the low range, low serum iron, high total iron-binding capacity, low transferrin saturation, and persistent anemia, which is more consistent with iron deficiency than iron overload at that point in the course. This finding supports continued evaluation for malabsorption, dietary restriction, blood loss, adherence issues, and the need for iron replacement, while also showing that the initial hyperferritinemia was likely reactive or liver-related rather than a straightforward iron-loading picture. If future results show elevated transferrin saturation or recurrent unexplained hyperferritinemia, HFE testing and hepatology input would remain reasonable [12,17].

Mild pancreatic atrophy on CT is also nonspecific. It may be incidental, age-related, related to prior or chronic pancreatic injury, associated with alcohol exposure, or reflect other gastrointestinal or metabolic conditions. Although pancreatic exocrine dysfunction can coexist with CD, CT evidence of mild atrophy alone does not establish clinically significant pancreatic disease. The revised manuscript therefore reports the finding without overstating its diagnostic significance.

### 3.5. Hypothyroidism, Thyroid Autoimmunity, and Celiac Disease

The coexistence of hypothyroidism and confirmed CD is clinically relevant. Meta-analytic evidence supports an increased prevalence of CD among patients with documented autoimmune thyroid disease [8]. In this case, however, thyroid autoantibody status and the duration of hypothyroidism were not available in the source record, so autoimmune thyroiditis cannot be confirmed from the reported data. The patient should therefore be described as having hypothyroidism, not confirmed autoimmune thyroid disease. Nevertheless, hypothyroidism increased the clinical rationale for targeted CD testing in the context of gastrointestinal symptoms, weight loss, anemia, osteopenia, arthralgia, and positive tTG-IgA.

The thyroid finding also limits attribution. The elevated baseline TSH was clinically meaningful and required levothyroxine adjustment. Subsequent normalization of TSH supports improved thyroid replacement, and improvement in somnolence, mood, and energy may have been partly related to thyroid correction. Future reports of similar cases should include baseline free thyroxine, thyroid antibody status, levothyroxine adherence, dose changes, and repeat TSH to better distinguish endocrine from gastrointestinal and psychiatric contributors.

### 3.6. Follow-Up, Applications, and Future Directions

The practical application of this case is a targeted screening approach. Primary-care physicians should not test every patient with anxiety or depression for CD. Testing becomes more appropriate when psychiatric symptoms are accompanied by gastrointestinal symptoms, weight loss, anemia, osteopenia, arthralgia, hypothyroidism or documented autoimmune thyroid disease, persistent liver-test abnormalities, infertility, neuropathy, dermatitis herpetiformis, or family history of CD [1,3].

Follow-up after a confirmed CD diagnosis should be more structured than advice to avoid gluten. Adult follow-up guidance emphasizes symptoms, dietetic support, nutritional status, serologic response, complications, adherence barriers, and long-term morbidity [18]. In this patient, the repeat tTG-IgA result and small-bowel histology directly addressed diagnostic certainty, while the follow-up iron studies, anemia, GGT, and thyroid tests clarified several competing issues. The most important ongoing steps are continued celiac serology, dietitian support, iron-deficiency management, thyroid monitoring, repeat liver assessment, bone-health review, and renewed discussion of gastroenterology/hepatology follow-up if abnormalities persist.

### 3.7. Strengths and Limitations

The main strength of this case is its realistic primary-care perspective. It illustrates how CD may be detected when psychiatric symptoms are embedded within a broader multisystem presentation. A second strength is that the report now includes objective follow-up data and histologic confirmation, which add diagnostic certainty beyond clinical improvement alone.

The limitations remain important. This is a single case report and cannot establish causality between CD and the psychiatric or hepatic manifestations. HLA-DQ2/DQ8 testing, thyroid autoantibodies, detailed alcohol quantification, viral hepatitis serology, autoimmune liver testing, complete repeat ALP/ALT/AST values, standardized mood scales, and patient-reported outcome measures were not available in the source narrative. Multiple interventions occurred simultaneously, including mirtazapine, levothyroxine adjustment, alcohol-reduction counseling, and GFD initiation. Therefore, clinical improvement cannot be attributed to the GFD alone. Finally, severe hepatic steatosis, residual GGT elevation, mild pancreatic atrophy, and evolving iron deficiency require independent follow-up and should not be over-attributed to CD.

## 4. Conclusions

This case supports targeted CD screening in adults presenting with anxiety or depressive symptoms when additional gastrointestinal, thyroid, musculoskeletal, hematologic, hepatic, nutritional, or bone-health clues are present. The patient had gastrointestinal symptoms, weight loss, arthralgia, hypothyroidism, anemia, osteopenia, marked liver-test abnormalities, severe hepatic steatosis, hyperferritinemia, and strongly positive tTG-IgA obtained before GFD advice. Small-bowel histology and repeat tTG-IgA testing confirmed CD and addressed the initial diagnostic uncertainty. At the same time, uncontrolled hypothyroidism, alcohol exposure, steatotic liver disease, mirtazapine initiation, thyroid correction, iron deficiency, and other follow-up findings mean that symptom improvement and liver-test improvement should still be interpreted cautiously. The core lesson is not that CD alone explains the entire presentation, but that careful primary-care assessment and follow-up can confirm CD while preserving a broad differential diagnosis and a clear monitoring plan.

## Figures and Tables

**Table 1:** Baseline findings and clinical interpretation.

Domain	Baseline or Diagnostic Finding	Reference Interval or Source	Clinical Interpretation
Hematology	Hemoglobin 118 g/L	120–160 g/L	Mild anemia; compatible with chronic disease, nutritional deficiency, liver disease, or CD-related malabsorption.
Iron/inflammation marker	Ferritin 1538 microg/L	20–300 microg/L	Marked hyperferritinemia; requires distinction between reactive elevation and iron overload.
Cholestatic liver marker, GGT	Alkaline phosphatase 445 U/L	30–145 U/L	Cholestatic or mixed liver-test abnormality; bone contribution also possible in osteopenia.
Alcohol/cholestatic marker	Gamma-glutamyl transferase 1479 U/L	8–35 U/L	Severe elevation; alcohol-related and hepatobiliary causes require consideration.
Hepatocellular marker	ALT 104 U/L	1–40 U/L	Hepatocellular component.
Hepatocellular marker	AST 256 U/L	8–32 U/L	Hepatocellular component; AST predominance may occur with alcohol-related liver injury.
Endocrine	TSH 21.99 mIU/L	0.2–4.00 mIU/L	Uncontrolled hypothyroidism; plausible contributor to fatigue, mood symptoms, and somnolence.
Celiac serology	tTG-IgA > 250 kIU/L	0.0–14.9 kIU/L	Strongly positive; supported CD in the setting of compatible symptoms and was later corroborated by histology.
Histology	Small-bowel biopsy during the diagnostic episode: moderate-to-severe crypt hyperplastic villous atrophy with increased intraepithelial lymphocytes; gastric biopsies: no significant pathology.	Surgical pathology report	Consistent with and supportive of histologically confirmed CD.
Radiographs	Mild osteoarthritis and mild osteopenia	No erosive arthropathy	Does not support inflammatory erosive arthropathy; osteopenia may be relevant to CD or other risk factors.
CT abdomen/pelvis	Severe diffuse hepatic steatosis; mild pancreatic atrophy; no malignancy	Imaging finding	Steatosis and pancreatic atrophy require integration into the differential; malignancy was not identified.

Abbreviations: ALT, alanine aminotransferase; AST, aspartate aminotransferase; CD, celiac disease; CT, computed tomography; GGT, gamma-glutamyl transferase; tTG-IgA, tissue transglutaminase immunoglobulin A; TSH, thyroid-stimulating hormone.

**Table 3:** Condensed timeline of the clinical course.

Time Point	Clinical Event	Interpretive Note
Index primary-care visit	Mood/anxiety symptoms, somnolence, reduced appetite, weight loss, bloating, diarrhea, flatulence, and polyarthralgia. Broad laboratory testing and radiographs were requested; mirtazapine 15 mg nightly was started at this visit.	The presentation was not treated as isolated psychiatric illness because systemic symptoms were present. Mirtazapine preceded the initial laboratory results.
Initial investigations before GFD advice	Mild anemia, marked hyperferritinemia, severe liver-test abnormalities, elevated TSH, and strongly positive tTG-IgA (>250 kIU/L) were identified. The tTG-IgA sample was obtained before gluten-free diet advice.	Findings supported CD but also highlighted endocrine, hepatic, alcohol-related, inflammatory, and iron-overload considerations. Timing of serology before dietary restriction strengthened interpretation.
Early management after initial results	GFD advice, alcohol-reduction counseling, levothyroxine adjustment, and community monitoring were initiated after the initial results were reviewed.	Management was multifactorial; clinical improvement could not be assigned to a single intervention.
Imaging and pathology during diagnostic episode	CT showed severe diffuse hepatic steatosis and mild pancreatic atrophy, with no malignancy. Small-bowel biopsy performed during the diagnostic episode showed moderate-to-severe crypt hyperplastic villous atrophy with increased intraepithelial lymphocytes; gastric biopsies showed no significant pathology.	Histology confirmed CD while imaging kept steatotic liver disease and alcohol-related injury in the differential. The record does not quantify day-to-day gluten exposure immediately before biopsy.
Approximately six months after initial management	Most symptoms had nearly resolved by patient report after GFD advice, alcohol-reduction counseling, levothyroxine adjustment, and mirtazapine treatment.	Clinical improvement was meaningful but multifactorial.
Longer-term objective follow-up (March–May 2026)	Repeat tTG-IgA remained positive but improved to 49.4 kIU/L. TSH normalized, GGT improved markedly but remained elevated, and iron studies showed iron deficiency with persistent anemia.	Repeat testing narrowed diagnostic uncertainty and identified ongoing monitoring needs.

Abbreviations: CD, celiac disease; CT, computed tomography; GFD, gluten-free diet; tTG-IgA, tissue transglutaminase immunoglobulin A; TSH, thyroid-stimulating hormone.

**Table 4:** Objective follow-up findings and remaining clinical implications.

Follow-Up Domain	Objective Follow-Up Finding	Interpretation and Remaining Need
Celiac disease activity and diagnostic certainty	tTG-IgA 49.4 kIU/L (reference <15.0), decreased from baseline >250 kIU/L but still positive. Small-bowel histology showed villous atrophy, crypt hyperplasia, and increased intraepithelial lymphocytes.	Findings support confirmed CD and partial biochemical response; persistent tTG-IgA positivity requires continued GFD adherence review and repeat serology.
Thyroid disease	TSH improved to 0.94 and 0.36 mIU/L; free T4 15.7 pmol/L.	Thyroid replacement was biochemically improved; symptom improvement may be partly thyroid-related. Continue routine thyroid monitoring.
Liver disease and steatosis	GGT improved to 104 U/L but remained elevated; INR 1.1. CT had shown severe diffuse hepatic steatosis.	Improvement is reassuring but incomplete and does not establish CD as the main cause of the baseline liver-test abnormalities. Repeat full liver enzymes and steatosis/alcohol/metabolic follow-up remain important.
Iron status and anemia	Ferritin 8–15 microg/L; iron 3–6 micromol/L; total iron-binding capacity 83–89 micromol/L; transferrin saturation 0.04–0.07; hemoglobin 95 g/L and later 110 g/L with microcytic features.	Follow-up pattern is more consistent with iron deficiency than iron overload at that time. Evaluate malabsorption, diet, blood loss, and need for iron replacement.
Nutrition, inflammation, renal function, and metabolic risk	Vitamin B12 375 pmol/L; vitamin D 65 nmol/L; CRP 2.9 mg/L; creatinine 46 micromol/L with eGFR 102 mL/min/1.73 m^2^; hemoglobin A1C 6.6%; lipase 114 U/L.	Several nutritional and inflammatory markers were reassuring; elevated A1C and mildly elevated lipase require routine clinical follow-up.
Mental health and patient-centered care	Symptoms improved after GFD advice, mirtazapine, thyroid correction, and alcohol-reduction counseling, which were introduced close together.	Improvement is clinically meaningful but multifactorial. Continue mood, sleep, appetite, alcohol-use, and dietary-adherence follow-up.

The table reports objective data available from follow-up testing and the clinical implications for ongoing care. Abbreviations: CD, celiac disease; GFD, gluten-free diet; GGT, gamma-glutamyl transferase; INR, international normalized ratio; tTG-IgA, tissue transglutaminase immunoglobulin A; TSH, thyroid-stimulating hormone.

## Data Availability

No public dataset was generated or analyzed for this case report. De-identified clinical details relevant to the case are contained within the article.

## Abbreviations

ACG, American College of Gastroenterology; ALP, alkaline phosphatase; ALT, alanine aminotransferase; AST, aspartate aminotransferase; CD, celiac disease; CT, computed tomography; EMA-IgA, endomysial immunoglobulin A; GFD, gluten-free diet; GGT, gamma-glutamyl transferase; HFE, homeostatic iron regulator gene; IEL, intraepithelial lymphocyte; IgA, immunoglobulin A; INR, international normalized ratio; tTG-IgA, tissue transglutaminase immunoglobulin A; TSH, thyroid-stimulating hormone.
